# High Entropy Alloys as Filler Metals for Joining

**DOI:** 10.3390/e23010078

**Published:** 2021-01-07

**Authors:** Dan Luo, Yong Xiao, Liam Hardwick, Robert Snell, Matthew Way, Xavier Sanuy Morell, Frances Livera, Nicholas Ludford, Chinnapat Panwisawas, Hongbiao Dong, Russell Goodall

**Affiliations:** 1 Department of Materials Science and Engineering, The University of Sheffield, Sir Robert Hadfield Building, Mappin St, Sheffield S1 3JD, UK; d.luo@sheffield.ac.uk (D.L.); lhardwick1@sheffield.ac.uk (L.H.); r.m.snell@sheffield.ac.uk (R.S.); mway1@sheffield.ac.uk (M.W.); xsanuymorell1@sheffield.ac.uk (X.S.M.); fslivera1@sheffield.ac.uk (F.L.); 2 School of Materials Science and Engineering, Wuhan University of Technology, Wuhan 430070, China; yongxiao@whut.edu.cn; 3 TWI Ltd., Granta Park, Great Abington, Cambridge CB21 6AL, UK; Nick.Ludford@twi.co.uk; 4 Department of Engineering, The University of Leicester, University Road, Leicester LE1 7RH, UK; chinnapat.panwisawas@leicester.ac.uk (C.P.); hd38@leicester.ac.uk (H.D.)

**Keywords:** high entropy alloys, joining, brazing, soldering, filler metals, alloy design

## Abstract

In the search for applications for alloys developed under the philosophy of the High Entropy Alloy (HEA)-type materials, the focus may be placed on applications where current alloys also use multiple components, albeit at lower levels than those found in HEAs. One such area, where alloys with complex compositions are already found, is in filler metals used for joining. In soldering (<450 °C) and brazing (>450 °C), filler metal alloys are taken above their liquidus temperature and used to form a metallic bond between two components, which remain both unmelted and largely unchanged throughout the process. These joining methods are widely used in applications from electronics to aerospace and energy, and filler metals are highly diverse, to allow compatibility with a broad range of base materials (including the capability to join ceramics to metals) and a large range of processing temperatures. Here, we review recent developments in filler metals relevant to High Entropy materials, and argue that such alloys merit further exploration to help overcome a number of current challenges that need to be solved for filler metal-based joining methods.

## 1. Introduction

**Figure 1:**
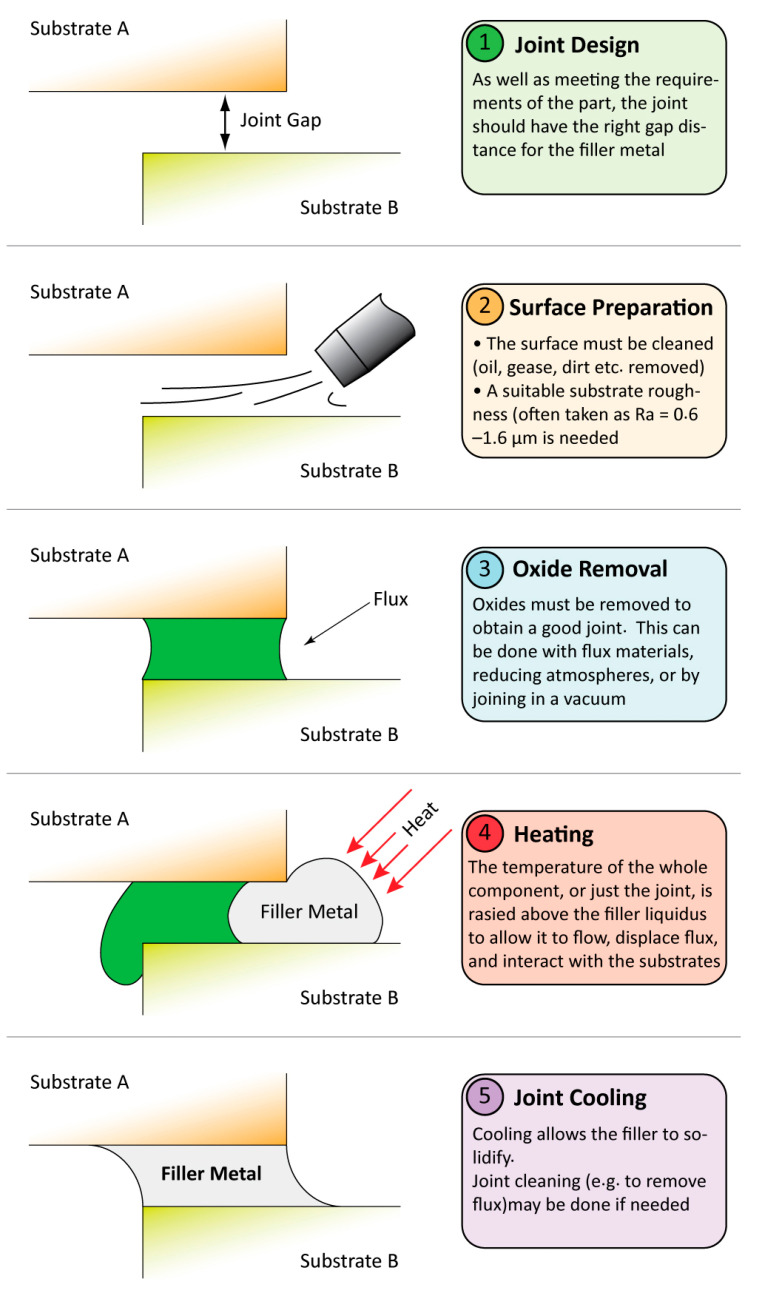
A schematic of the five main stages in joining using a filler metal process. Note that the details of these steps may vary considerably in different applications, and the pictorial representation here does not depict the details of all processes.

Joining is vital in the assembly of components when products cannot, due to size or material requirements, be fabricated as a single piece. Joining technology, applied in industries as diverse as electronics, aerospace, automotive, and energy, offers a wide range of different techniques, each with particular characteristics, which will determine their suitability for a certain application. Among these, brazing and soldering offer the principal advantage that they are capable of forming a metallurgical joint between widely dissimilar substrate materials (the parts being joined) with minimal modification of those materials [1,2,3]. In both brazing and soldering processes, a molten filler metal is used to form a metallurgical bond between two (or more) components (the convention is for the two methods to be distinguished by a watershed of 450 °C). It is important to note that in brazing and soldering, the filler metal is a more distinctive alloy than the filler metals used to make up joint volume in a number of fusion welding processes; in that case, the filler metal usually has a similar composition and melting point to the materials being joined, while in soldering and brazing, the filler metal is the only part of the joint that melts. It is heated above its liquidus temperature and allowed to wet (and often flow between) the components it is being used to join. Once in the correct position, the filler is allowed to cool to a solid state, forming a metallurgical bond adhering the components together [4,5,6]. The key steps in joint formation by these techniques are shown in Figure 1.

Filler metals therefore play a significant role in brazing and soldering, and are likely to have a very different composition to the materials they are used with. Selection of filler metals depends on a multitude of factors, e.g., the parent materials, service conditions, joint design and required filler metal form, the brazing process and temperature, as well as various legal requirements and regulations which may apply to specific situations [7,8,9,10]. As a result, although selection criteria will vary with application, they often include melting characteristics (the solidus and liquidus temperatures and the melting range), wetting (which can be more complex for ceramic-ceramic joining [11] or metal-ceramic joining [11]) and flow. Previous developments for use with different materials have led to a large number of different filler metal alloys being developed; there are more than 80 brazing filler metals, and more than 60 solders listed in standards, with many more being sold commercially as specialist compositions [12,13,14] (indeed, an estimate of the number of brazing filler metals in use worldwide numbers almost 800 [15]).

To achieve better properties for joining, new filler material alloys and combinations of materials have attracted more and more attention, such as metallic glasses [16] and the use of metal foams to manage residual stresses [17], or as reinforcements [18,19], but research has only recently begun to investigate the potential for High Entropy Alloys (HEAs) to be used as filler metals. HEAs (also known as multi-principal component alloys, or complex concentrated alloys) are a set of alloys that form a simple structure despite having many components and no dominant element [20,21,22]. They generally contain five or more elements, each between 5 at. % and 35 at. % [23], though these limits are not strictly established and the term is still applied to some alloys not following these rules [24,25,26]. HEAs have been suggested to have several unique behaviors and unusual properties [27], some of which could be of use for filler metals. For example, the high mixing entropy possible with multiple components could promote the formation of a random solid solution over the brittle phases, which reduce the mechanical properties in many joints where they occur (such as in some instances of nickel brazing), due to the interaction between filler and substrate [28,29]. This could also allow the inclusion of high levels of elements in filler alloys to control the melting temperature, wetting, and flow behavior, without inducing brittle phases. It may also be possible to include elements in filler metals that would, on initial assessment, appear to be incompatible with the parent materials, and moreover, the multicomponent nature could mediate the transition in a joint between dissimilar materials. However, investigation of HEAs as filler metals is as yet very limited. This paper reviews the results achieved to date and makes the case for further development of HEAs as filler metals, focusing in on a number of specific cases where there are application challenges that current filler metals cannot meet.

### 1.1. Existing Filler Metals

While it is not in the scope of this review to discuss existing fillers in general, understanding some of the common alloys, and in particular where these have some similarities to HEAs, is useful, and a brief overview is given.

#### 1.1.1. Solders

Modern solders are frequently Sn-based; these are very versatile, and exist in numerous compositions, often based around eutectic systems, such as Sn-Ag, Sn-Zn or Sn-In. In electronics manufacturing, multiple reflows (the operation in which soldered joints are formed in the processing of electronics) are required to achieve 3-dimensional integration of circuits and packaging where there is multiple stacking of chips. Hence, the availability of solder materials with different melting ranges is important to avoid repeated melting and solidification of pre-existing joints during reflows.

In former times, Sn-Pb solders were popular, as they show favorable melting temperature, excellent wettability, high reliability, and low cost. Now, however, the health and environmental effects of Pb make such solders unacceptable in most applications, and they have in the most part been replaced by the Sn-Ag-Cu series solders (sometimes known as “SAC” solders) [30,31,32]. A typical Sn-Ag-Cu series solder composition is Sn-3.0Ag-0.5Cu (wt.%), with a melting point of 217 ℃. The main challenges facing Sn-Ag-Cu series solders are controlling the growth of the interfacial intermetallic compound layer, preventing the formation of Sn whiskers and inhibiting the formation of Kirkendall cavities.

**Figure 2:**
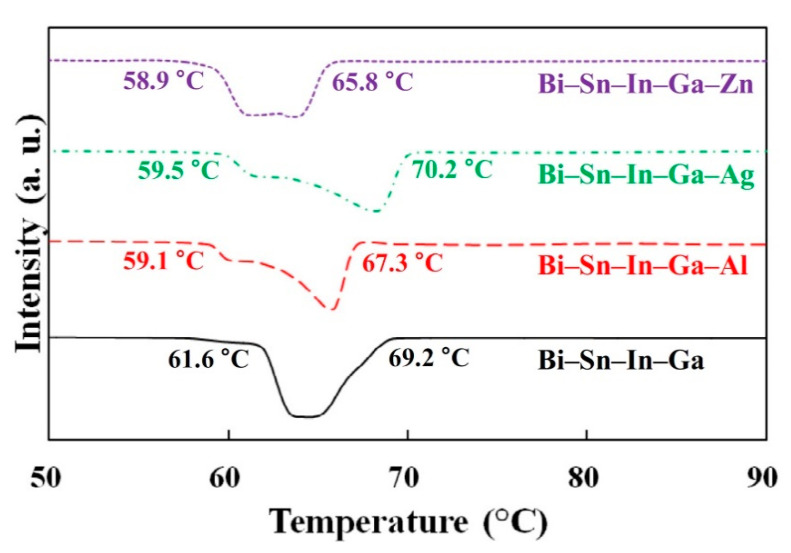
Differential Scanning Calorimetry (DSC) data from some High Entropy Alloy (HEA)-like solders, indicating the effect of the elemental composition on the melting point. Reprinted from [34], with permission from Elsevier.

To overcome such limitations, other solders, such as those based on the Sn-In eutectic, have been explored. These exhibit excellent ductility, flexibility, and high thermal and electrical conductivity, and are regarded as promising materials for soldering heat sink components for flexible devices [7]. However, their relatively low mechanical strength and melting temperature still need improvement for more effective use in areas such as flexible interconnection applications, and this has led to the exploration of some complex compositions. For instance, alloys explored include some close to HEA compositions including quaternary Sn-Bi-In-Zn [33], and Bi-Sn-In-Ga and quinary alloys formed from this by adding Zn, Ag, and Al [34]. Figure 2 shows Differential Scanning Calorimetry tests on these alloys, indicating the effect of the fifth element on the melting point.

#### 1.1.2. Brazing Filler Metals

Brazing covers a very wide range of temperatures, and the joining of many different materials. As a result, there is a wide range of filler metals available (for a full account see [2]). These include Al-based alloys, which are the lowest melting point common brazing filler metals. There are also Ag-based alloys, which wet a wide range of common engineering materials and are quite widely used; in these, to reach the lowest melting temperature (similar to what was formerly achieved with Ag-Cd alloys), compositions towards the middle of the Ag-Cu-Zn ternary are explored, resulting in compositions that approach multicomponent alloys, such as Ag34 Cu36 Zn28 Sn2 and Ag30 Cu28 Zn21 Cd21 (wt.%), existing within the standards.

There are also fillers designed for high temperature use, based on nickel or precious metals. Among the many different alloys used in these classes, a number have near-equiatomic combinations of elements, including Pd21 Mn31 Ni48, Au30 Ni36 Pd34, and Ni44 Fe35 Cr11 Si7 B2 Cu1 (wt.%).

## 2. Development of HEAs as Filler Metals

Although none of the standard compositions of filler metals meet the stricter definition of High Entropy Alloys, there are some, highlighted above, that approach these, suggesting that exploration of the capability of such alloys to function as fillers is worth examining, and attention has turned in this direction. Note that, in what follows, we use the convention suggested by Pickering and Jones [27], and name HEAs in order of increasing atomic number of the elements, unless there is a non equiatomic composition and clearly more dominant elements in the composition than others; this results in some alloys being renamed, in that the elements are reordered from how they are given in their original references.

### 2.1. Application of Reported HEAs as Fillers

**Figure 3:**
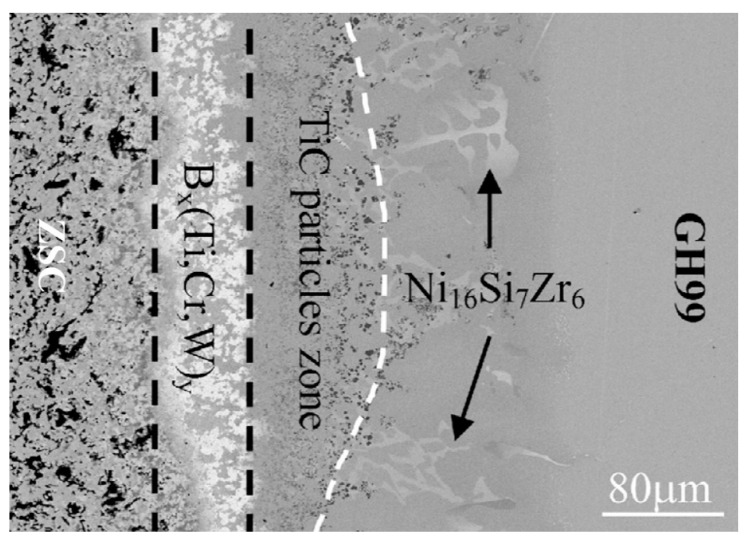
A cross section through a joint formed between ZSC and GH99 superalloy, using CrFeCoNiCu and Ti foils, at 1180 °C, 60 min. Reprinted from [35], with permission from Elsevier.

**Figure 4:**
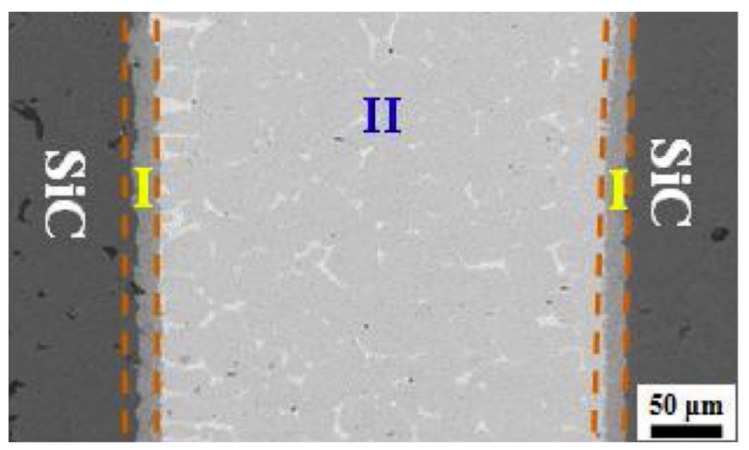
A cross section through a joint formed between two pieces of SiC, using CrFeCoNiCu alone as the filler, at 1180 °C, 60 min. (**I**) Denotes the reaction layers and (**II**) the main joint seam. Reprinted from [39], with permission from Elsevier.

Zhang et al. [35] took the known HEA equiatomic CrFeCoNiCu high entropy alloy and made a composite, active brazing filler by combining it with pure Ti foil to braze ZrB2-SiC-C to GH99 superalloy. They concluded that the high mixing entropy of the Ti/CrFeCoNiCu composite filler helps to retain the activity of Ti and Cr during bonding, and that this changes the interfacial reaction involving Ti and Cr. The main matrix of the joint consists of a solid solution phase, and this contains TiC and borides (a structure suggested to be responsible for the good joint strength seen) (Figure 3). Bridges et al. [36] used the same alloy in the laser brazing of Inconel 718 superalloy, achieving a 220 MPa maximum shear strength with an effective brazing temperature of 1165 °C [36]. Tillman et al. [37] used Ge, Sn, and Ga as low level additions to CrFeCoNiCu. These elements had a MPD effect, and reduced the melting range of the alloy to allow it to join Ni-based superalloys. They also used the eutectic high entropy alloy N0.73CrFeCoNi2.1 to join Crofer 22 APU to Hf-metallized yttria-stabilized zirconia in Solid Oxide Fuel Cells (SOFC) and sound joints were obtained [38]. Wang et al. successfully brazed SiC to itself [39] and to ZrB_2_ [40], also using CrFeCoNiCu as the filler, in which Cr_23_C_6_ formed due to reaction at the interface, Figure 4, showing the capacity of this alloy to act as an active filler. High shear strength was observed, suggested to arise from the formation of solid solution in the brazed joint. It is notable that Cr is not often used as an active addition in fillers, and greater understanding of the potential for different elements in multicomponent alloys to act in this way would be an important basis for better design of active filler metals.

### 2.2. Development of New HEA Compositions as Fillers

Some new HEA compositions have been developed to improve the filler metal performance. Some, like Gao et al. [41,42] are variants of other HEA alloys; they developed and demonstrated the use of Fe_5_Co_20_Ni_20_Mn_35_Cu_20_ for brazing the Ni-Cr-Fe based Alloy 600. They achieved a maximum joint shear strength of 530 MPa with a 90-min hold time at 1200 °C [42]. This is comparable to shear strengths observed in some tests with conventional materials.

Some new compositions are arrived at by using HEA principles with elements used in filler metals. Novel Ag-based HEAs have been sought to reduce the costs of brazing fillers that use precious metals [43], and the approach has been applied to design low temperature brazing fillers [44]. The approach has also been used for solders. Pu et al. [33] studied a very low temperature HEA SnBiInZn solder with a melting temperature of 80 °C. This solder has good wetting ability, and the joint is strong enough (19–28 MPa) for electronic packaging technology. Hardwick et al. introduced a filler based on NiCrFeGeB [45]. It is noteworthy that this was not an equiatomic composition, but the design started with an equiatomic alloy and was modified using CALPHAD to adapt the expected properties to those required. Sharma et al. [46] reviewed the issues in metal-ceramic brazing and solutions to overcome those issues using various fillers, and suggested the approach of HEA brazing filler metals.

To illustrate more precisely the existing work developing HEAs as filler metals, and to highlight where future opportunities may lie, we discuss some of these particular examples in more detail in the following sections, focusing on the motivation for using HEAs to address different needs. These comprise the development of new fillers to reduce reliance on expensive or difficult to source elements; widening the operating temperature range of fillers (both those that can operate at higher temperatures without brittleness due to intermetallics and those that can fill the gap at intermediate temperatures between soldering and brazing); fillers for extreme environments and solders with improved reliability in service.

## 3. Reducing Reliance on Undesirable Elements

From the examination of the standard compositions, it is clear that filler metals are very diverse. While offering great variety in properties, this means that fillers use elements that may not be the most desirable from the point of view of cost (e.g., Au, Ag, Pd). As well as the high value of these elements, as commodity metals, the volatility in their price can make the cost of filler metals far less predictable than would be desirable. HEAs may be able to help here, as in such alloys no element dominates, so they may provide a route to form viable alloys with much reduced concentrations of expensive elements. If unexpected properties in combinations can be found, they may even provide a route to eliminate these high cost elements all together.

**Figure 5:**
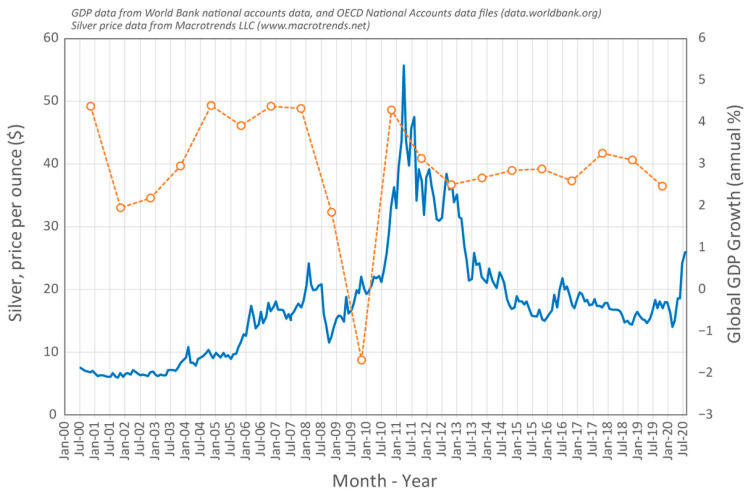
Historical data for the commodity price of silver (in USD per ounce), which impacts the cost of purchasing the metal and the value of the alloys it is used in. The data for global GDP growth are shown for comparison, to illustrate the correlation with one of the factors that contributes to price rises for Ag.

To illustrate this, we can briefly explore the development of novel fillers in the silver brazing alloy family. The price of silver shows the volatility with global economic conditions discussed above; data for the last 20 years of commodity silver prices, with global GDP growth for comparison, are shown in Figure 5. In times of economic disturbance, such as currently, the price of silver can increase by a factor of three or more.

Silver-based alloys are a diverse and popular range of brazing filler metals. The silver content offers attractive brazing properties, such as lower melting points, enhanced flow, and improved material versatility compared to filler metals with no silver [2]. Many existing fillers are based on the Ag-Cu-Zn ternary; the Ag-Cu phase diagram has a significant eutectic that is useful in reducing the melting points of the filler metal. Zinc also lowers the melting temperature but the formation of brittle intermetallics does limit the quantity that can be included [47] (overcoming such limits is potentially another advantage of the HEA approach, discussed in a later section).

To try and find new alloys, Snell [43] performed a large-scale search, using simple parameters to gauge the likelihood of successful alloy formation from the work of Zhang et al. [23]. This used a python script to systematically trial a large number of compositions (3,141,528) from 336 alloy systems using nine elements (Ag, Cu, Zn, Sn, Mn, Ni, Co, In, and Ga). The starting elements were selected based on the currently used Ag-Cu-Zn ternary and other elements, which can feature in these filler metals. The process worked by systemically discarding alloys with unfavorable predicted properties and optimizing on the remaining alloys. Properties were evaluated using the likelihood of HEA formation (calculating parameters using the equations from Zhang et al. [23], and evaluating on the limits applied there), melting temperature (using a eutectic approximation [43]), and cost.

The compositions discarded at an early stage showed that tin and indium were largely incompatible as part of a HEA structure, mainly due to large atomic size differences with the other atoms. Tin and indium could have had an effect of melting temperature reduction, and so the only remaining element under consideration that would have this effect was zinc. The optimization therefore focused on increasing the zinc content. The most promising system, by far, was the four-component Ag-Cu-Zn-Mn system. The addition of manganese and alteration of the Ag-Cu ratio from the common amount in silver fillers meant that the predicted results could accommodate much more zinc than in the conventional alloys, and this had the additional effect of leading to a reduction in the amount of silver required (although silver still remains one of the major components of the alloy).

**Figure 6:**
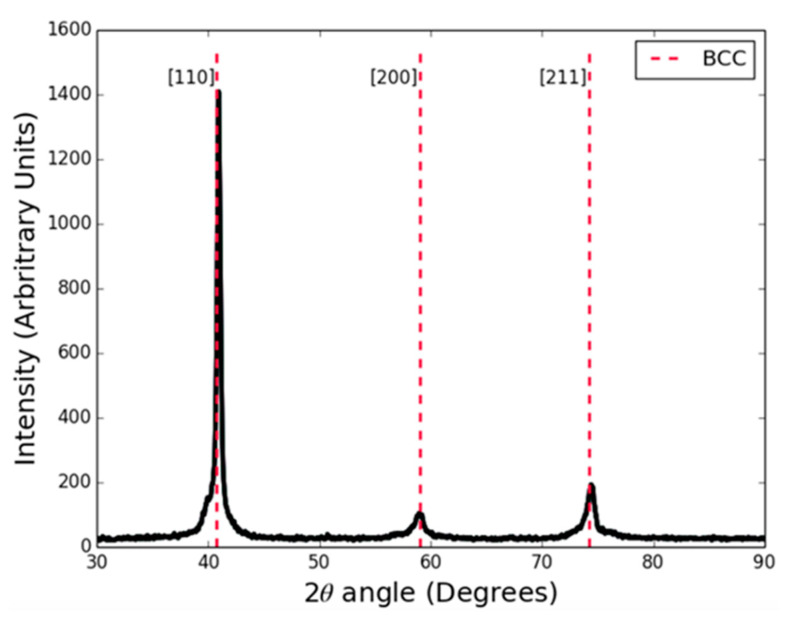
XRD pattern for the HEA brazing alloy (Ag37-Cu14-Zn45-Mn4 at. %). The scan was taken using Bruker D2 Phaser and a Cu Kα source. The peaks identified show a single phase BCC structure.

The investigation into the Ag-Cu-Zn-Mn system covered many compositions, including many that did not meet all of the rules often used to define HEAs. The results showed that with careful calibration of the elements, the properties (atomic size difference and enthalpy of mixing) could be adapted to allow much greater quantities of zinc to be included in the alloy without the formation of intermetallics being predicted. One of optimum alloys in this regard was Ag37-Cu14-Zn45-Mn4 at. %. The alloy was produced from 99.9% or better purity elements by induction melting and examined in XRD (Bruker D2 Phaser and a Cu Kα source), Figure 6, and, to the resolution of the system used, shows only a single phase Body Centered Cubic (BCC) structure.

For this particular alloy, and alloy system, the benefits were unfortunately offset by the drawbacks. The high zinc content suppresses the melting points and reduces the alloy cost. However, in order to achieve such high zinc content, the Ag-Cu ratio has to be moved away from the eutectic and some additional manganese is required. This offsets the benefits and results in a filler metal of roughly similar properties as the non-HEA equivalent.

While this alloy is not therefore attractive as a commercial product, the principle of using HEAs and the design rules that have been developed for them to find new filler metals outside existing composition ranges (for example using less of a certain element) appears to be effective.

## 4. Widening Service Temperature

### 4.1. Increasing Service Temperature, Avoiding Brittle Phases

Some of the most demanding conditions that brazed joints may be employed in is the high temperatures found, for example, within jet engines and nuclear pressure vessel containers. In these cases, the filler must have high oxidation/corrosion resistance, good creep resistance, or be able to maintain its mechanical properties and microstructure stability. This is a difficult combination to achieve, especially with the requirement that the filler should melt before the materials being joined; however, the ability to use materials at these temperatures would allow, for example, the yield in jet engines to be increased [14,48,49]. The next generation of jet turbines will need to work at higher temperatures (up to 1200 °C), and weight reduction is highly desirable. This leads to consideration of new materials such as ceramic matrix composites, which would need to be joined to superalloys or titanium alloys, presenting new joining challenges [50,51,52].

**Figure 7:**
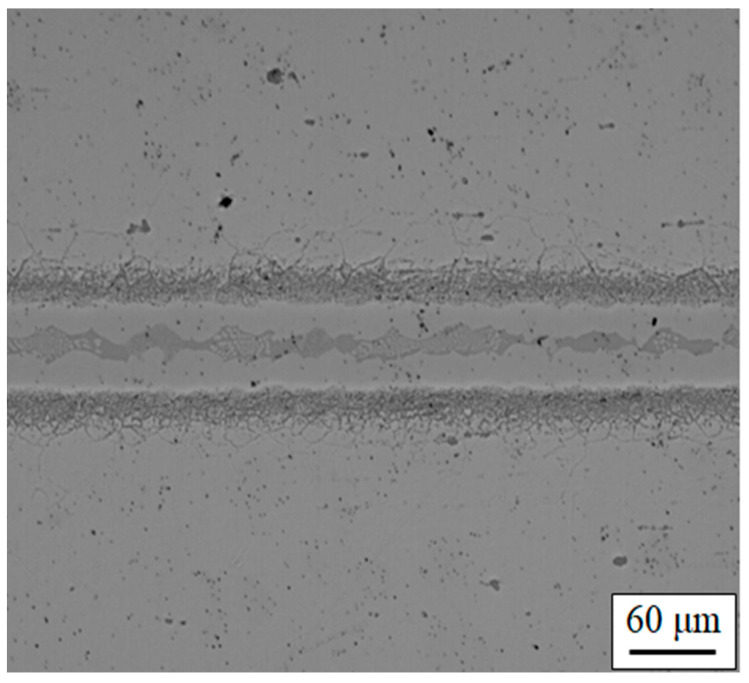
Inconel 718 alloy joined by standard AWS BNi-2 filler.

Already for the existing materials a number of fillers have been developed. The standard fillers, can be classified into two main groups depending on the microstructure, which results from two main approaches to achieving the goals. The fillers in one of these groups are based on precious metals (Au, Pd) and are usually solid solutions with additions of nickel and copper. These alloys derive their good oxidation resistance from the precious metal content, and often also show good mechanical properties at high temperatures [14], but, as discussed in Section 3, bring increased cost. The other approach produces a group of alloys based on nickel or nickel-chromium, which make use of alloy systems with eutectic compositions. These alloys normally see the addition of an element exhibiting a eutectic composition in the binary phase diagram with Ni (normally boron, silicon, or phosphorus), and thus acting as a melting point depressant (MPD). They have good corrosion resistance at high temperatures due to the chromium content, but the MPD element, which is necessary to reduce the melting point to a level where it can be used as a filler, usually results in the formation of intermetallic phases (borides, silicides, or phosphides), which can be brittle and may reduce corrosion resistance locally, in both the joint region and where diffusion into the base metal has occurred. In some approaches, extended high temperature treatments are used to disperse the MPD elements by diffusion; this dilutes the MPD effect, and thus imparts a high melting point in the joint. A typical joint between two superalloy pieces with this type of filler is shown in Figure 7.

As HEAs are suggested to result in greater than expected chance of finding the formation of solid solutions at the expense of intermetallics, they may be a route to allow the incorporation of MPDs to convert a very high melting temperature alloy into one that could be used for brazing (either reducing the heat treatment required or allowing it to result in a more temperature-resistant joint). Indeed, the nickel superalloys currently used in high-temperature applications already have compositions closer to that of HEAs than many other alloy systems. They typically have a high atomic percentage of primary alloying elements Cr, Fe, or Co, in addition to other alloying elements such as Ti, Al, Nb, W, Ta, Re, and Mo; indeed, this increasing complexity of nickel alloys is increasing the challenges for the brazing process. It is possible that HEAs may also be able to offer suitable properties, for example high levels of strength and good oxidation resistance at high temperatures [53]. It has also been reported that some existing HEAs have good creep resistance, and at the same time, some active elements can be added to increase the wettability during the joining process, and improve the quality of the final braze [39].

Exploration of HEAs as brazing filler metals for joining superalloys is currently at an early stage, and much initial work has looked at the use of transition metal HEAs similar to or based around the Cantor alloy (CoCrFeMnNi) [37]. Gao et al. recently demonstrated the development and application of a Fe_5_Co_20_Ni_20_Mn_35_Cu_20_ multi-principle element alloy for brazing the Ni-Cr-Fe based Alloy 600 [42]. They achieved a maximum joint shear strength of 530 MPa with a 90-min hold time at 1200 °C. Bridges et al. [36] used the same composition alloy in the laser brazing of Inconel 718 superalloy, achieving a 220 MPa maximum shear strength with an effective brazing temperature of 1165 °C. A CoFeCrNiCu HEA has been used to join SiC, which as a ceramic material requires an active filler metal; in this process, the Cr was found to have acted as the active element [39]. The solid solution phase found in the joint after brazing at 1180 °C for 1 h was reported to have resulted in increased shear strength as compared to that achieved by the established Ag-Cu-Ti filler metal (although the brazing temperature required to achieve this is substantially higher than would be required for processing with Ag-Cu-Ti). Nb was used in a Ni-17.2Cr-17.2Co-17.2Fe-12.5 (in at. %) filler metal for brazing yttria-stabilized zirconia to Crofer 22 stainless steel, achieving joint strength of approximately 103 MPa and reportedly twice the strength achieved in the use of a typical Ag-Cu-Ti filler metal [54] (again, the brazing temperature required for the former was significantly higher than required for the latter). It is also worth noting that in the mechanical testing of such ceramic to metal joints the sample size may be an important factor; smaller samples more commonly explored in the laboratory could result in higher strength through reduction in the residual stress accumulated during processing.

In the studies above, brazed joints were obtained without the use of MPD elements, and so undesirable phases identified were those already either present in the HEA filler metal, or due to interaction with the base material, such as in the case of the ceramic joints. Due to this lack of MPD, however, the brazing temperatures required for these systems were very much at the high end of the capability of most standard equipment and therefore higher than most temperatures that are commonly used in practical settings (especially for ceramic to metal joining). On the one hand, the composition of the HEAs trialed is typically single-phase FCC with solid-solution strengthening, which is attractive mechanically. On the other hand, for practical and cost purposes, the high required brazing temperature could be prohibitive. Compositions might therefore be sought that reduce the brazing temperature required. However, even though alloy liquidus temperature tends to decrease towards the equiatomic composition, it is often not sufficient in achieving a sufficiently low brazing temperature. The solution to this may lie in moving away from equiatomic composition HEAs. This idea has already seen much research for developing eutectic/dual-phase HEAs [55], or HEAs with secondary phases [56], deliberately with the aim of enhancing the strength of the HEA (though promotion of BCC or other ordered phases), while retaining the ductility from the FCC phase. Such a concept could be applied to also reduce the alloy liquidus temperature to that suitable for use as a brazing filler metal.

**Figure 8:**
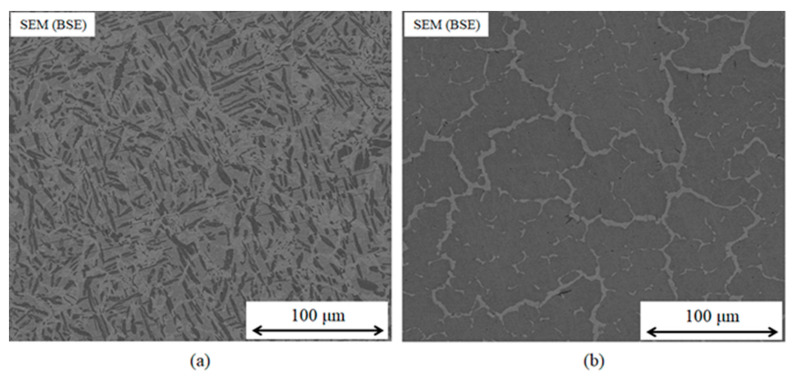
SEM (BSE) micrographs of as-cast (**a**) equiatomic NiCrFeGe, and (**b**) optimised Ni, Fe-rich, Cr-lean NiCrFeGe alloys. Significant reduction in the concentration of the dark contrast Cr,Ge-rich intermetallic phase is observed in (**b**) as compared to (**a**), resulting from the Thermo-Calc modelled composition optimization towards increased FCC solid solution molar content.

Another route would be to retain the approach of including a MPD element, and to rely on the suppression of ordered compounds in HEAs to avoid intermetallic formation. This approach has been studied by Tillman et al., who investigated the addition of the elements Sn, Ge, and Ga to equiatomic CrFeCoNiCu [37]. However, even at Ga content of up to 16.66 at. % the liquidus of the filler metal was only reduced to 1259 °C, though this was sufficient to braze Mar-M 247 at 1275 °C for 30 min, achieving a shear strength of 388 ± 73 MPa even with a relatively high thickness of 200 μm [37]. Hardwick et al. [57] also used this approach, with Ge as the primary MPD element (example microstructures are shown in in Figure 8) along with a 2.5 at. % B addition, forming NiCrFeGeB. This filler metal was found to possess a liquidus temperature of 1055 °C, and was used to vacuum braze Inconel 718 superalloy at 1100 °C for 15- and 60-min hold times. Following the latter, the minor B addition was largely diffused out from the joint, and an isothermally solidified joint was achieved, consisting of a non-equiatomic Ni-Cr-Fe-Ge solid solution phase. Despite this, there were some indications that the balance between strength and ductility had not been optimized. The joint shear strength achieved was 296 MPa, compared to 476 MPa using AWS BNi-2 filler metal, which displayed a greater degree of plastic deformation prior to failure.

### 4.2. Intermediate Temperature Fillers

**Figure 9:**
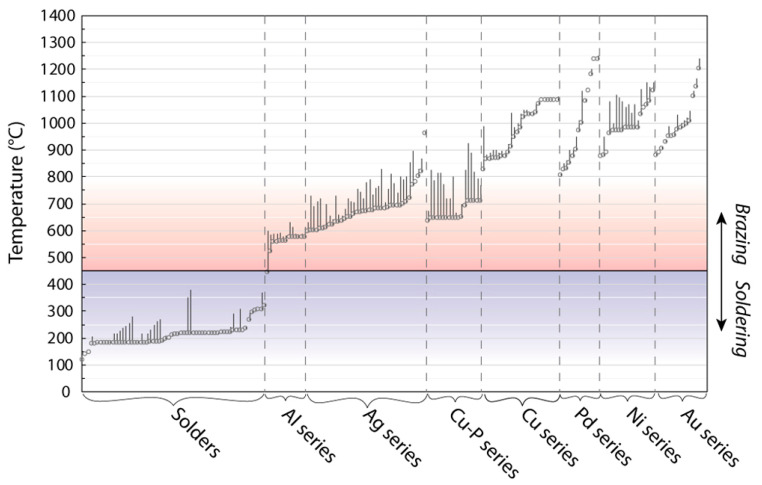
The melting characteristics of standard soldering and brazing filler metals, split into the families used in the standards. The plotted point is the solidius, and the vertical line represents the melting range until the liquidus temperature is reached. The low number of available fillers around 350–500 °C is clear. Data for brazing filler metals are from ISO: 17672 and for solders from BS EN ISO 9453.

As mentioned above, brazing and soldering are divided by the temperature watershed of 450 °C. This is set in a temperature region poorly occupied by the standard filler metals, as shown in Figure 9. It is clear that certain temperatures are well served by fillers (noting, however, that more than just the correct melting temperature is needed, and a filler metal will also have to wet and be compatible with the metals it is to bond), but that there is a gap between approximately 325 °C and 550 °C. This reflects the former absence of a strong need for such fillers, but with new applications now arising that require the use of filler metals with intermediate melting temperatures that fall in this range, this sparsity of usable fillers becomes a challenge which requires addressing.

The draw towards this temperature range can occur for various reasons. For the lower temperature fillers (the solders) increasing the temperature of use may permit joints that operate at higher temperatures. For brazing filler metals, reduction in joining temperature may allow the bonding of materials with lower melting points, or temperatures at which their properties become degraded. Narrowing the gap can also allow additional steps in step brazing or soldering (where a component is assembled with a series of joints at decreasing joining temperature). We will discuss in more detail how efforts from both solders and brazing filler metals have sought to enable processing in this zone.

#### 4.2.1. Higher Temperature Solders

Much research has been done on low-temperature Pb-free solders, leading to many options being available [58], but the solders that can operate at high temperature are limited. These would be attractive for step soldering technology, flip–chip connections, solder ball connections, and the bonding of semiconductor devices onto substrates due to environments due to various applications, such as in high-temperature electronics (HTE) [59] and relocating compact electronic devices of automotive-engine compartments [60]. For efficient manufacture and assembly of electronic components, a melting temperature range within 270–350 °C is desirable [58]. This is established as solders with solidus temperature above 270 °C can work at higher service temperatures or withstand the peak temperature of a second soldering treatment in step soldering, and the liquidus temperature needs to be below 350 °C to avoid thermal degradation of the polymers commonly used in the substrate [61] (step soldering is used in other areas too, such as heavy truck radiators made of copper; this also demands solders of different melting ranges to avoid the remelting of seams [62]). Common solders for high temperature applications that aim to meet these requirements are mainly based on Pb–Sn, Au–Sn, Au–Ge, Zn–Al, Zn–Sn, Bi–Ag, and Sn–Sb alloys [61].

Nevertheless, it is likely that further development toward higher temperatures will be needed. Higher operating temperatures or new materials (functional materials for example) may need higher temperature solders [63], and researchers have attempted to create these. Xing et al. [62] studied the effect of Sn added to a Zn-2Cu-1.5Bi high-temperature solder, when used to join Cu substrates. It was found that a melting range of 390−400 °C was obtained with a 3 wt% addition. However, these minor additions are only able to modify the properties to a certain extent. To try and develop solders with melting range around 400–500 °C (to join Al-based composites in electronics packaging) Li et al. investigated Au-Ag-Ge and Ag-Cu-Sb [64]. Alloys meeting the requirements included Au-10.6Ag-10.5Ge and Ag-33.5Cu-20.5Sb with melting ranges of 401–441 °C and 483–488 °C, respectively. These ranges, and those of some other high-temperature solder alloy systems, can be found in Table 1. While none of these alloys are HEAs, it is clear that some of the higher temperature alloys, involving more elements such as Au, Ag, or Ge, which are less common in low temperature solders, have higher levels of the additional elements. It may therefore be the case that searching further for alloys of this type among HEA compositions may be fruitful, either in terms of searching combinations of elements that are little explored, or taking advantage of the multiple alloying elements to find ways of incorporating higher melting point elements into solders to increase their melting temperature.

#### 4.2.2. Reduced Temperature Brazing Filler Metals

Brazing is usually defined as occurring above 450 °C, and many fillers melt at much higher temperatures, producing thermally resistant joints. There is however a need to have more filler metals that melt at temperatures down to 450 °C, and perhaps even lower. An example of this is with some of the high strength grades of aluminum alloy (e.g., 7000 series), where the amount of alloying additions that are necessary to achieve these strengths reduce the melting temperature to levels where brazing with existing fillers is not possible. As seen in Table 1, the development of the higher temperature solders is often driven by interest in joining aluminium alloys, and this could also motivate reduced temperature brazing fillers.

Another case is the joining of temperature sensitive functional materials, such as skutterudite thermoelectrics [77,78]. Thermoelectric generators (TEGs) may have a future role in energy efficiency [64,77,79,80], for example to recapture waste heat in automotive exhausts. A material with good performance is CoSb3-based n-type skutterudite thermoelectric [77], which is easier to use and more environmentally friendly than PbTe [81]. To achieve its optimum power production, the temperature needs to be 550 °C or above [77,78], but temperatures that exceed 620 °C may lead to sublimation of Sb from the material and thus degrade it [82]. To survive operation, but not damage these skutterudite thermoelectrics on processing, the optimal melting temperature range for the filler metal would be T = 550–620 °C. Initially, examination of the standard fillers (Figure 9) suggests 2 silver-based and 10 aluminum-based fillers will meet this requirement, but they either contain the banned element cadmium [2], react with the thermoelectric to form detrimental compounds [83], or otherwise see excessive diffusion of filler elements into the thermoelectric, compromising performance [84].

**Figure 10:**
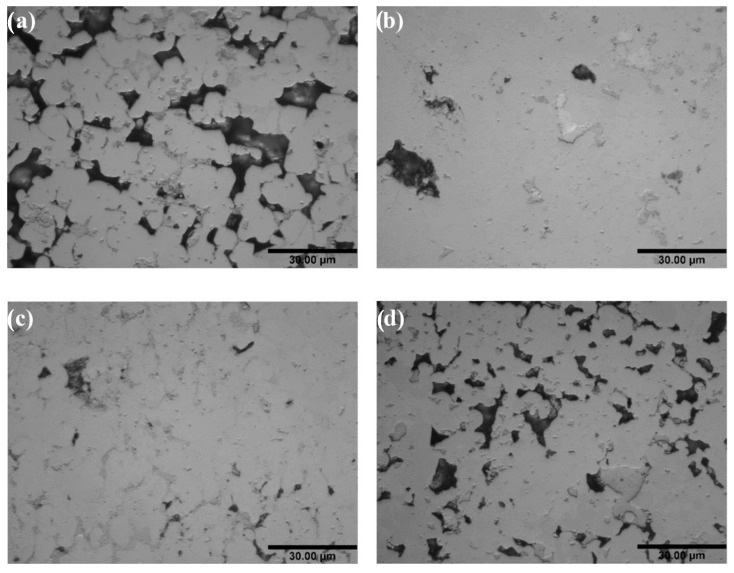
Optical micrographs of the microstructure of each of the four alloy systems at 100x magnification. ZnGaCu-(AuSn) (**a**), ZnGaCu-Sn (**b**), ZnGaCu- (AuBi) (**c**), and ZnGaCu-Bi (**d**). All four show the presence of multiple phases. Note there is also porosity as these observations of the alloys were made after initial alloying and casting, before preparation of joints.

Attempts have been made to use HEA design approaches to develop fillers for this specific application [44]. This makes use of the ability in HEAs to combine a number of elements with different characteristics into an alloy and to promote mixing. A ZnGaCu-(AuSn) composition and related alloys result, Figure 10, which show a number of favorable properties.

## 5. Fillers for Extreme Environments

**Figure 11:**
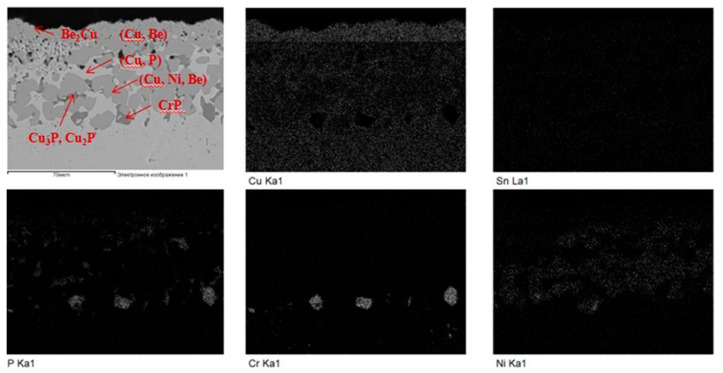
SEM image of a joint formed between Be and CuCrZr using a Cu-9.1Ni-3.6Sn-8.0P filler (STEMET 1101), and the EDS compositional maps. Many different phases are identified as indicated on the SEM image. Reprinted from [92], with permission from Elsevier.

Brazing is frequently used where the materials are dissimilar, where the components are complex with different part sizes, and also where the operating conditions will be severe. An example that imposes all of these conditions is in the plasma facing components (PFC) of the International Thermonuclear Experimental Reactor (ITER) [85,86]. In the PFC, brazed joints can be found in the first wall, limiter, baffle, and divertor components [87,88]. There are different armor material candidates for the PFC, including beryllium and tungsten (in both cases to be joined with Cu alloy heat sink materials) [86,89,90]. These joints must have good physical and mechanical properties, and maintain these after the irradiation and cyclic heat fluxes without degradation. Be has some advantages as an armor material (low atomic number, which can significantly improve the plasma performance [86], relatively high thermal conductivity, low tritium retention, and low activation in the reactor [86,91]). However, Be reacts with most elements at elevated temperatures, leading to the formation of brittle intermetallic phases, which makes brazing difficult and reduces joint strength and ductility [87]. The reactivity of Be with Cu is especially high, and intermetallic phases can form at 350~400 °C [87]. Only few elements commonly included in fillers (Al, Si, Zn, Ag, and Ge) do not form stable beryllides below 760 °C [92]. An example of the typical microstructure of a joint formed between Be and CuCrZr using a Cu-9.1Ni-3.6Sn-8.0P filler (STEMET 1101) is shown in Figure 11, indicating the different phases present.

To solve the problems caused by complex phase formation, the reaction time when brazing Be and Cu is reduced by the use of induction brazing, which gives more rapid local heating and showed much better joint quality than conventional furnace brazing when using a Ag-Cu brazing alloy [5,93]. However, silver-based fillers are not permitted in ITER due to transmutation to cadmium during neutron irradiation [87], so silver-containing fillers (such as 72Ag–28Cu, Cu–15Ag–5P, in wt.%) have been replaced by Cu-based filler metals (such as Cu-Ni-Sn-P) [92]. Diffusion barriers are also used in the brazing process to eliminate the formation of intermetallics [86]. These diffusion barriers must be compatible with the parent materials and not exhibit extensive alloying during the process [86]. Soft or compliant layers may also be used to reduce the residual stresses caused by the difference of the coefficient of thermal expansion (CTE) [87]. CTE differences are also the challenge with one of the main competitors to beryllium as an armor material, tungsten. The large difference in CTE and elastic modulus across W/Cu joints creates large stresses at the interface for a conventional flat tile geometry, possibly requiring different interface shapes [87].

The development of new filler metals could also be an effective way to solve these challenges. For use in nuclear fusion application, compatibility with the extreme environment is the first criteria [94]. Some HEAs have been shown to be resistant to damage of the type encountered in nuclear environments [95] and could offer a good opportunity for such extreme conditions. For brazing Be/Cu elements compatible with Be could be added into the filler metals at large amounts, while avoiding the formation of stable detrimental intermetallics, which could produce a stronger joint and also avoid the need for diffusion barriers. The wide compositional ranges that could be achieved without fundamentally altering the alloy could also adjust the CTE of the filler to act as an intermediate layer in the W/Cu joints. The approach could also be suitable for other candidate materials. Active elements could be also added to improve wetting of the filler on carbon composites. Therefore, new HEA alloys could be a promising approach in such extreme conditions.

## 6. Improved Service Reliability of Solders

**Figure 12:**
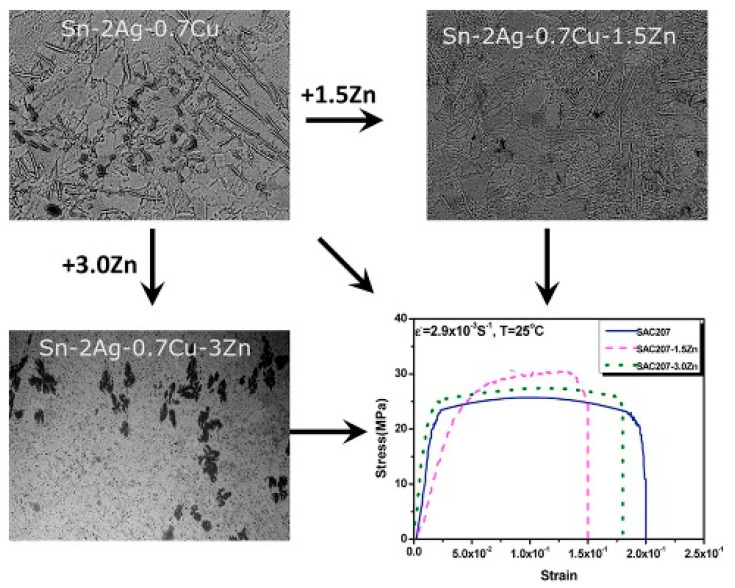
The effect of Zn content on the microstructure and tensile strength of Sn-2Ag-0.7Cu. Reprinted from [107], with permission from Elsevier.

Solders are extensively used in the electronics industry, and with the continual miniaturization of electronic devices, there is increasing need for better performing soldered joints. The main current alloys used for this application are the SAC solders (Sn-Ag-Cu), which have generally suitable mechanical and physical properties though the in-service reliability of these joints is generally lower than with the former Sn-Pb series solders [96,97,98,99]. Improving the reliability of such joints is a current focus for development, and the desired improvements span several aspects:

**(a) Improved solder strength:** In general, the reliability of soldered joints is greatly influenced by the properties of the solder. It has been reported that a large amount of Ag_3_Sn intermetallic compound (IMC) present in SAC solders (which occurs at high Ag levels, around 3 wt.%) causes inhomogeneous stress distribution and reduction of reliability [30,100]. However, reducing Ag also leads to the decrease of mechanical properties, including resistance to thermal fatigue and creep [100,101]. Different alloying elements have been added to improve the mechanical properties, including Ni, Bi, Zn, Sb, and Ga [102,103,104,105,106] and it has been found that small levels of additions can suppress the formation of large Ag_3_Sn plates in SAC solders, and improve the microstructure and mechanical properties [32]. For example, small Zn additions (e.g., 1.5 wt%) to a Sn-2.0Ag-0.7Cu solder refined Ag_3_Sn and Cu_6_Sn_5_ particles, and also promoted the formation of small (Cu, Ag)_5_Zn_8_ intermetallic particles, which improved the yield strength and ultimate tensile strength, at the cost of decreased ductility [107], as shown in Figure 12.

**Figure 13:**
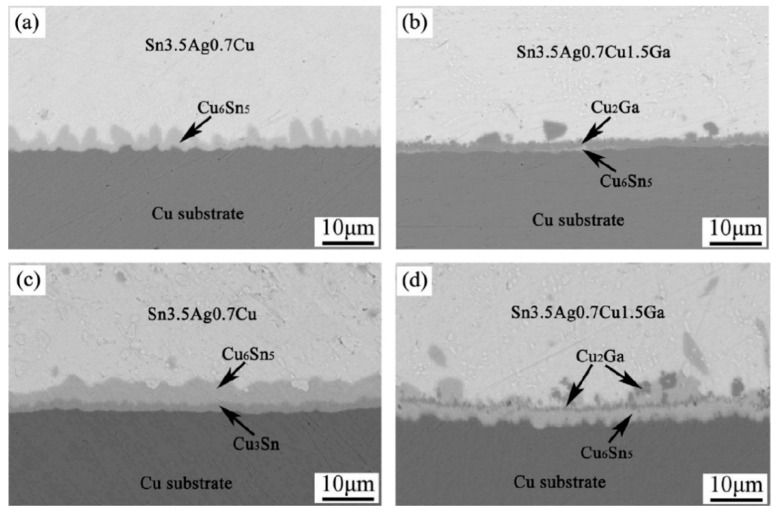
Morphologies of as-reflowed (**a**) Sn-Ag-Cu/Cu and (**b**) Sn-Ag-Cu-Ga/Cu interface, (**c**) Sn-Ag-Cu/Cu and (**d**) Sn-Ag-Cu-Ga/Cu interface aged at 180 °C for four days. Reprinted from [105], with permission from Elsevier.

**(b) Inhibit excessive growth of the interfacial IMC layer:** Generally, intermetallic compounds (IMC) at the interface are harmful. For instance, in Cu/Sn/Cu soldered joints, rapid growth of brittle IMCs containing Cu_6_Sn_5_ and Cu_3_Sn can reduce joint properties. Nickel is commonly added into low-Ag SAC solders to suppress the growth of Cu_3_Sn and improve strength when bonding Cu; however, this addition also increases the melting temperature and melting range [108]. An addition of Bi can reduce the thickness of Cu_6_Sn_5_ at the interface and increase both the tensile-shear strength and elongation [108] and Pb is also known to be effective, but is not preferred due to toxicity [109]. The addition of Ga both decreases the melting point of SAC solder, and also inhibits the growth of Cu_6_Sn_5_ and Cu_3_Sn IMC layers [105], as shown in Figure 13. This results in a shear strength increase by about 4 MPa to 33 MPa in the case of a Sn-3.5Ag-0.7Cu-1.5Ga/Cu soldered joint compared to the same alloy without Ga [105]. However, all of these additions are relatively small, and a large amount of hard and brittle phases result when solders contain high additions of further elements.

**(c) Avoid the formation of Kirkendall cavities:** The formation of Kirkendall cavities is attributed to the unequal diffusion flux of different elements across the interface. With continual progress in miniaturization of electronic devices, solder joints are subjected to growing current density and temperature during service, and under these conditions electromigration and thermal diffusion of elements may occur, resulting in the formation of Kirkendall cavities. The addition of some alloying elements can avoid this in Sn solder, including sulfide forming elements such as Mn, Zn, and Ag, which are thought to work by trapping impurities at interface [110,111]. As discussed in (b), the addition of Ni, and also Zn, can suppress the growth of Cu_3_Sn at the Sn solder/Cu substrate interface and thus avoid the formation of cavities that arise due to this [112,113,114].

**(d) Inhibit the formation of whiskers:** Interconnection failure, resulting from the growth of whiskers (especially in Pb-free, Sn-based soldered joints), which interconnect and provide a short circuit, has attracted great attention [115]. Previous research has shown that the addition of alloying elements such as Nd [116], Ga [116], Zn [117], and Ce [118] into lead-free solders can inhibit the growth of Sn whiskers, by encouraging the formation of certain stable compounds.

Solutions to both (a) and (b) above could be sought by using the potential ability of HEAs to favour the formation of solid solution over the more highly ordered intermetallic compounds. By seeking alloys that approach closer to equiatomic than have been explored before, it may be possible to find ones that can include silver without producing the phases that reduce strength, and compositions that produce less of a reaction layer with the substrate than those already known. It may also be possible to draw on the high strength reported in some HEAS to improve the base properties of the solder.

The issues described in (c) and (d) could also be addressed by new HEA alloys, especially if the suggested “sluggish diffusion effect”, limiting the rate at which elements diffuse through a HEA, does really occur in all alloys.

## 7. Conclusions and Future Perspectives

It is clear that the potential effects encountered in HEAs, and the wide compositional space that the approach opens up for exploration, are attractive to address a number of challenges presently faced by filler metals. Research has begun in this area, and with intriguing discoveries such as the potential of active bonding with some existing HEA compositions, and the development of new HEAs as filler metals with compositions outside the usual HEA classes, the future for alloys under this broad definition as fillers seems bright.

In order to further this field, better understanding of HEA behavior is, however, required. Diffusion is an important aspect of many situations in which fillers are used, but is not fully understood for HEAs, with debate over the postulated sluggish diffusion effect. Other properties important for fillers, such as the wetting behavior or the flow of the alloys when liquid have not been investigated for HEAs specifically. It may be that these processes occur in ways entirely consistent with other alloys, or it may be that the multicomponent nature introduces other effects, which could be exploited to improve bonding or widen the range of materials to which the methods can be applied.

Advanced characterization techniques may also have a role to play in understanding the alloys developed. For example, in situ neutron diffraction has been used to determine the evolution of lattice strain, stacking fault probability, and dislocation density in a FeCoCrNi high-entropy alloy fabricated by powder metallurgy [119]; the same technique could be applied to investigate features during the process of brazing or soldering, such as formation of intermetallics at the interface or stress determination (especially where dissimilar materials are used) [120]. Modelling could offer another opportunity. Ab initio methods, such as density functional theory (DFT) have been used to investigate HEAs, and are also of use in joining, for example to study the influence of Ti on the interfacial bonding between SiO_2_ and an Ag filler containing Ti [121], and the effect of Cr, B, and Si on the interfacial bonding between diamond and a Ni-Cr-B-Si filler Zhang et al. [122].

With such approaches, the objective would be to achieve sufficient understanding that finding a HEA or HEA-like alloy suitable as a filler could be the result of a design process, rather than a serendipitous discovery.

## Figures and Tables

**Table 1:** The solidus and liquidus temperatures of various reported intermediate temperature fillers.

Fillers	Melting Range (°C)	Substrate(s)	Target Application	Ref.
Zn-4Al	381–409	Cu	Electronics	[65]
	381–412	Cu, Ni, Ag	Electronics	[66]
Zn-2Cu-1.5Bi-7Sn	387–393	Cu	Electronics	[62]
Zn-15Al	388–447	5052 Al	Not specific	[67]
Zn-5Sn-2Cu-1.5Bi-0.1RE	390–395		Functional materials	[68]
Zn-2Cu-1.5Bi-3Sn	391–400	Cu	Electronics	[62]
Zn-1.5Al-2.0Re	400–410	AZ31B/Al6061	Not specific	[69]
Au-10.6Ag-10.5Ge	401–441	Al-SiC composite	Electronics	[64]
Au-13.8Ag-10.5Ge	410–450	Al-SiC composite	Electronics	[64]
Ag-13.3Cu-42.4Sb	423–429	Al-SiC composite	Electronics	[64]
Mg-28.7Al	438–460	AZ31B	Not specific	[70]
Zn-15Al-0.3Zr	445 (liquidus)	6061 Al/304SS	Automotive/aerospace	[35]
Mg-65.1In-6.4Zn-0.7Al	449 (liquidus)	AZ31	Not specific	[71]
Al-38.7Mg-9.2Zn	451–469	AZ31B	Not specific	[72]
Zn–22Al	451–488	5052 Al	Not specific	[67]
Mg-64.6In-1.2Zn-0.8Al	471 (liquidus)	AZ31	Not specific	[71]
Al-8.4Si-20Cu-10Ge-0.1Re	479–514	Al/Ti-6Al-4V	Aerospace/chemical	[73]
Ag-33.5Cu-20.5Sb	483–488	Al-SiC composite	Electronics	[64]
Al-8.4Si-20Cu-10Ge	489–513	Al/Ti-6Al-4V	Aerospace/chemical	[73]
Al–6.5Si–42Zn–0.12Sr	493–520	6061 Al	Automotive; electrical connections	[74]
Al-18Ag-20Cu-5Si-0.2Zn	494–515	AA2219/AISI 304	Not specific	[75]
Al-7Si-20Cu-2Sn-1Mg	501–522	3003 Al	Heat exchangers	[76]
Al-9.6Si-20Cu	523–535	Al/Ti-6Al-4V	Aerospace/chemical	[73]

## Data Availability

No new data were created or analyzed in this study. Data sharing is not applicable to this article.
